# The Role of PET/CT in Breast Cancer

**DOI:** 10.3390/diagnostics13040597

**Published:** 2023-02-06

**Authors:** Bawinile Hadebe, Lerwine Harry, Tasmeera Ebrahim, Venesen Pillay, Mariza Vorster

**Affiliations:** 1 Department of Nuclear Medicine, College of Health Sciences, University of KwaZulu Natal, Private Bag X54001, Durban 4001, South Africa; 2 Inkosi Albert Luthuli Central Hospital, Durban 4001, South Africa

**Keywords:** breast cancer, PET

## Abstract

Female breast cancer has surpassed lung cancer as the most commonly diagnosed cancer worldwide, with an estimated 2.3 million new cases (11.7%), followed by lung cancer (11.4%) The current literature and the National Comprehensive Cancer Network (NCCN) guidelines state that ^18^F-FDG PET/CT is not routine for early diagnosis of breast cancer, and rather PET/CT scanning should be performed for patients with stage III disease or when conventional staging studies yield non-diagnostic or suspicious results because this modality has been shown to upstage patients compared to conventional imaging and thus has an impact on disease management and prognosis. Furthermore, with the growing interest in precision therapy in breast cancer, numerous novel radiopharmaceuticals have been developed that target tumor biology and have the potential to non-invasively guide the most appropriate targeted therapy. This review discusses the role of ^18^F-FDG PET and other PET tracers beyond FDG in breast cancer imaging.

## 1. Introduction

Female breast cancer (BC) has the fifth highest mortality rate [1] with death rates for female breast and cervical cancers being considerably higher in developing versus developed countries (15.0 vs. 12.8 per 100,000 and 12.4 vs. 5.2 per 100,000, respectively). In females, it accounts for one in four cases and for one in six deaths [2]. 

Effective management of breast cancer requires accurate diagnosis and determination of the extent of the disease to select the most effective treatment approach [3]. Breast cancer is very heterogenous and is characterized by different pathological features, with distinct responses to treatment and differences in long-term patient survival [4]. Approximately 70% of BC express the estrogen receptor (ER), and the majority of ER+ cancers also express the progesterone receptor (PR). Collectively, ER+ cancers are classified as luminal, which are further subclassified based on their HER2 status and proliferation rate as Luminal A (ER/PR+, HER2−, Ki67+ < 20%), Luminal B (ER/PR+ < 20%, HER2−, Ki67+ ≥ 20%), and triple positive HER2+ B2 (ER/PR+, HER2 overexpression). Other molecular subtypes are the HER2 enriched (ER−/PR−/HER2+) and ER−/PR−/HER2− (or triple-negative breast cancer (TNBC)) [5].

In general, ER expression seen in luminal A and B responds well to hormonal therapy and is associated with excellent long-term survival [5,6]. HER2 overexpression is associated with a poor prognosis; however, its presence predicts a positive therapeutic response to anti-HER2 drugs. TNBC is highly invasive and has the poorest prognosis because it is not sensitive to endocrine therapy or molecular targeted therapy [5,6,7]. Therefore, chemotherapy is the main systemic treatment, but the efficacy of conventional postoperative adjuvant chemoradiotherapy is poor. Molecular analyses are performed as part of the routine pathological examination; unfortunately, these are limited by sampling errors and predict tumor response to antihormonal therapy correctly in only 50–60% of the patients [5]. Moreover, discordant receptor expression between primary tumor and metastatic lesions occurs in 18–55% of the patients, and it is impractical to biopsy all the lesions in patients with stage IV disease. Thus, molecular imaging has become vital in breast cancer as it allows non-invasive visualization of the biological markers and potential therapeutic targets in both the primary and metastasis; however, this is not routinely used. 

With the growing interest in personalized medicine, including molecular targeted therapy, immunotherapy, and theranostics, the role of molecular imaging in breast cancer has evolved. PET/CT imaging has an emerging role in the identification of specific potential targets in the tumor-microenvironment and selecting patients who might benefit from novel molecular-targeted therapies, thus maximizing the therapeutic effect and minimizing toxicity. In this review, we will discuss the role of PET/CT imaging in the diagnosis, staging, prognostication, recurrence assessment, radiotherapy planning, restaging, and treatment response of patients with BC and in selecting patients eligible for novel targeted therapies.

## 2. The Role of ^18^F-FDG PET/CT in Breast Cancer

^18^F-Fluorodeoxyglucose (FDG) is a glucose analog transported via glucose transporters into the cells and phosphorylated by hexokinase [8]. FDG follows the same pathway as glucose during the first enzymatic reactions in the cells, but because FDG lacks a hydroxyl group at the C-2 position, it is not metabolized further and is physically trapped in tumor cells at a rate proportional to glucose utilization [8].

Malignant cells show higher glucose metabolism and increased glycolytic activity as a result of increased glucose transporter (GLUT-1) expression and increased levels of hexokinase and phosphofructokinase compared to non-malignant cells [9]. This high glycolytic activity eases the detection of malignant cells using ^18^F-FDG PET imaging [8]. The role of ^18^F-FDG PET/CT in breast cancer is in diagnosis, staging, prognosis, treatment response evaluation, radiotherapy planning, and detection of recurrence.

### 2.1. Diagnosis

To date, mammography is the standard of reference for the detection of primary breast tumors. Either mammography or ultrasound detects the changes in the morphology of the breast tissue [10]. Due to the high cost and low sensitivity for detection of small lesions (<5 mm), therefore the use of PET/CT for diagnosis of early-stage breast cancer is limited by its low spatial resolution PET/CT is not routine for early diagnosis of breast cancer according to the current literature and the National Comprehensive Cancer Network (NCCN) guidelines [3]. 

The sensitivity and specificity of PET/CT for the diagnosis of breast cancer varies depending on the histological subtype and the size of the tumor from 48–96 and 73–100%, respectively [11,12,13,14]. Many studies have shown high sensitivity (greater than 90%) and variable specificity of FDG PET for the detection of large and palpable primary breast tumors [8,9,10,11]. However, FDG PET imaging has low sensitivity (<50%) for the detection of sub-centimeter breast cancers [8] due to the limited spatial resolution of PET. In addition, some tumors demonstrate low FDG avidity, such as ductal carcinoma in situ, lobular carcinoma, or tubular carcinoma, as well as grade 1 breast cancer, and may not be detected on FDG PET [8,9]. FDG uptake is also lower in well-differentiated ER+/PR+ tumors than in ER-/PR- tumors [10]. On the other hand, invasive carcinomas show higher uptake compared to ductal carcinoma in situ (DCIS) [11,12,13,14]. Among luminal tumors, FDG uptake is higher in luminal B than in luminal A tumors [15].

Unfortunately, FDG is not specific for malignancy and also demonstrates increased uptake in inflammatory and infectious lesions. Cancer detection is also limited by a significant number of physiologic processes, such as brain glucose uptake or muscle uptake [11]. Furthermore, FDG also accumulates in benign conditions, such as infection, fibroadenoma, ductal adenoma, inflammatory granulomatous mastitis, and fibrocystic changes resulting in a lower specificity [9].

Some authors have suggested dual-time imaging, that is, obtaining a second series of PET images centered on the breast approximately 2 h after FDG injection in order to improve specificity [9,16]. FDG uptake increases with time in malignant lesions, while most inflammatory lesions show uptake that remains stable or decreases over time. Newer imaging technologies in PET/CT imaging, such as total-body PET imaging, have an improved sensitivity which allows for imaging at much later time points post radiotracer injection (up to 5–6 half-lives); therefore, patients can be scanned at 2 h post radiotracer injection, which may potentially improve specificity for detection of malignant lesions [16]. Nonetheless, dual-time imaging is time-consuming, and its usefulness is yet to be confirmed in a large series [9].

It is necessary to explore focal breast uptake detected incidentally during an FDG examination performed for other indications with mammography and ultrasound imaging and possible biopsy because of the high risk of malignancy. A meta-analysis by Bertagna et al. reviewing incidental FDG uptake detected in the breast during PET or PET/CT conducted for other indications demonstrated a high pooled risk of malignancy of 60% in incidentally detected breast uptake upon histological examination [13]. 

### 2.2. Staging

Accurate initial evaluation of disease spread is important for treatment selection and prognostication in breast cancer. The initial staging work-up includes many conventional imaging modalities, such as mammography, magnetic resonance mammography, plain chest radiography, bone scintigraphy, and breast, axillary, and liver ultrasonography [10,11]. Studies comparing the impact of FDG PET/CT for initial staging of therapy naïve breast cancer with conventional imaging tools have shown that FDG PET/CT has the added benefit of the detection of extra-axillary (infraclavicular, supraclavicular, and internal mammary) lymph nodal metastasis and occult distant metastasis. Additional findings revealed by PET/CT may prompt a considerable change in the staging and management of 25% and 18% of breast cancer patients, respectively [13,14,15,16].

Nevertheless, the exact clinical stage at which PET/CT can be performed with well-balanced cost-effectiveness is uncertain [9]. The 2022 National Comprehensive Cancer Network guidelines suggest that PET/CT scanning should be performed for patients with stage III disease or when standard staging studies yield non-diagnostic or suspicious results [2]. The NCCN guidelines further state that “PET/CT may be helpful in identifying unsuspected regional nodal disease and/or distant metastasis in locally advanced breast cancer in addition to standard studies and is not recommended in the staging of clinical stage I, II, or operable III (T3 N1) breast cancer, due to its high false-negative rate for the detection of lesions that are small (<1 cm) and/or low-grade disease, the high rate of false-positive scans in patients without locally advanced disease.” However, meta-analyses suggest that a non-negligible proportion of even stage 1 disease (11%) and stage II disease (20%) is upstaged by FDG PET/CT imaging. Another study reported upstaging to stage IV of 14% (27/196) in patients with findings of unsuspected distant metastasis, including 13% of stage IIB (10/79) and 22% of stage III disease [17].

#### 2.2.1. Axillary Lymph Node Staging

The presence of lymph node metastasis is the single most important prognostic factor for treatment planning in breast cancer. A meta-analysis and systematic review of 25 studies investigating the accuracy of PET/CT in comparison with sentinel lymph node biopsy (SLNB) showed that the performance of PET/CT was inferior to SLNB; however, the high specificity of PET/CT in axillary lymph node assessment of 94% shows that FDG PET may have a role under certain circumstances [18]. For example, a positive lymph node on PET/CT in a patient with a high suspicion of advanced disease may guide direct axillary lymph node (ALN) dissection and spare the patient an SLNB. 

Nevertheless, PET/CT cannot replace staging by the sentinel lymph node biopsy due to the inability to detect early axillary lymph node disease and micrometastases [8]. Therefore, because of the limited sensitivity of PET/CT in comparison with SLNB, SLNB is still the method of choice to diagnose ALN involvement [9].

#### 2.2.2. Distant Metastasis Staging

It is well established that the prevalence of distant metastases is directly related to the stage of breast cancer at diagnosis [8]. As the staging of the disease increases, the possibility of having distant metastases also increases [11]. Although FDG PET/CT performs poorly compared with mammography, ultrasound, breast MRI, and axillary nodal pathologic evaluation for early disease, it becomes increasingly useful for advanced disease, particularly for the detection of extra-axillary nodal and distant metastases [17]. Thus, FDG PET/CT should be aimed at patients at a higher risk of metastatic disease. 

Patients with stage III disease have the highest rate of detection of extra-axillary lymph node metastases and distant metastases at initial presentation, and, therefore, initial staging with FDG PET/CT imaging would have the greatest clinical impact in this group of patients [13]. The detection of previously unsuspected distant metastases has a considerable clinical impact by upstaging patients to stage IV disease, which changes patient management from curative-intent therapy by surgery with or without neoadjuvant therapy for locoregional disease to palliative systemic therapies [17,18,19,20]. Patients diagnosed with stage IIB disease on mammography, ultrasound, or breast MRI have lower rates of detection for unsuspected more-advanced disease and upstaging [18,19] and may therefore be considered for FDG PET/CT based on the Ki67 index and the molecular subtype of the tumor. Rates of upstaging in stage I and IIA disease are very low, and FDG PET/CT is not indicated in these patients [2]. 

The common sites of distant metastasis in breast cancer are the bones, lungs, liver, and brain. Conventional imaging studies for detecting distant metastasis include chest radiographs, liver ultrasound, contrast-enhanced CT of the chest and abdomen, bone scintigraphy, and MRI [8]. In a meta-analysis of eight PET/CT studies (748 patients), Hong et al. reported a sensitivity of 96% and a specificity of 95% for the detection of distant metastasis by FDG PET/CT [21]. This was confirmed by Sun et al., who reported a sensitivity of 99% and specificity of 95% in a meta-analysis of six studies (609 patients) [22].

Bone is the most frequent site of distant metastases in BC, accounting for about 65% of patients with distant metastases [11]. Because of its high sensitivity and affordability, bone scintigraphy remains the standard procedure for the detection of bone metastases in breast cancer patients [23]. Although FDG PET/CT outperforms CT or bone scintigraphy for detecting lytic or mixed bone metastases and bone marrow lesions, FDG PET is less sensitive for purely sclerotic bone metastases [17,18,19,20,21,22,23,24]. For this reason, some clinicians still perform bone scintigraphy even after patients have undergone FDG PET/CT imaging. Nonetheless, non-FDG-avid sclerotic bone metastases are often detected on the CT component of the hybrid PET/CT procedure. Several authors have demonstrated that FDG PET/CT is more accurate than scintigraphy for the depiction of bone metastases. In a meta-analysis of seven studies, Rong et al. compared bone scans and FDG PET/CT (668 patients in total), FDG PET/CT outperformed bone scintigraphy with a sensitivity of 93% and specificity of 99%, whereas bone scintigraphy had a sensitivity of 81% and specificity of 96% [24]. Importantly, the grade of the tumor should be borne in mind when selecting the best imaging modality for staging breast cancer [25]. A recent study by Iqbal et al. comparing FDG PET/CT with conventional imaging in 74 patients with grade 1–2 ER + BC cases reported that FDG PET inadequately staged 22.9% of grade 1–2, ER + BC cases. Therefore careful assessment of the CT component is vital, especially when FDG PET/CT is negative; also, bone scintigraphy may be considered in these patients [26].

It is important to note that there are several FDG-avid false positive findings that should be identified and distinguished from malignancy to increase the specificity of FDG PET/CT [14]. These include bone marrow activation from colony-stimulating factors, Paget disease, fractures, avascular necrosis, iatrogenic injuries, benign neoplasms, and systemic inflammatory diseases. These should be distinguished from malignancy by correlation with clinical history, evaluation of the distribution of FDG uptake, and findings on the corresponding CT images [14].

**Figure 1:**
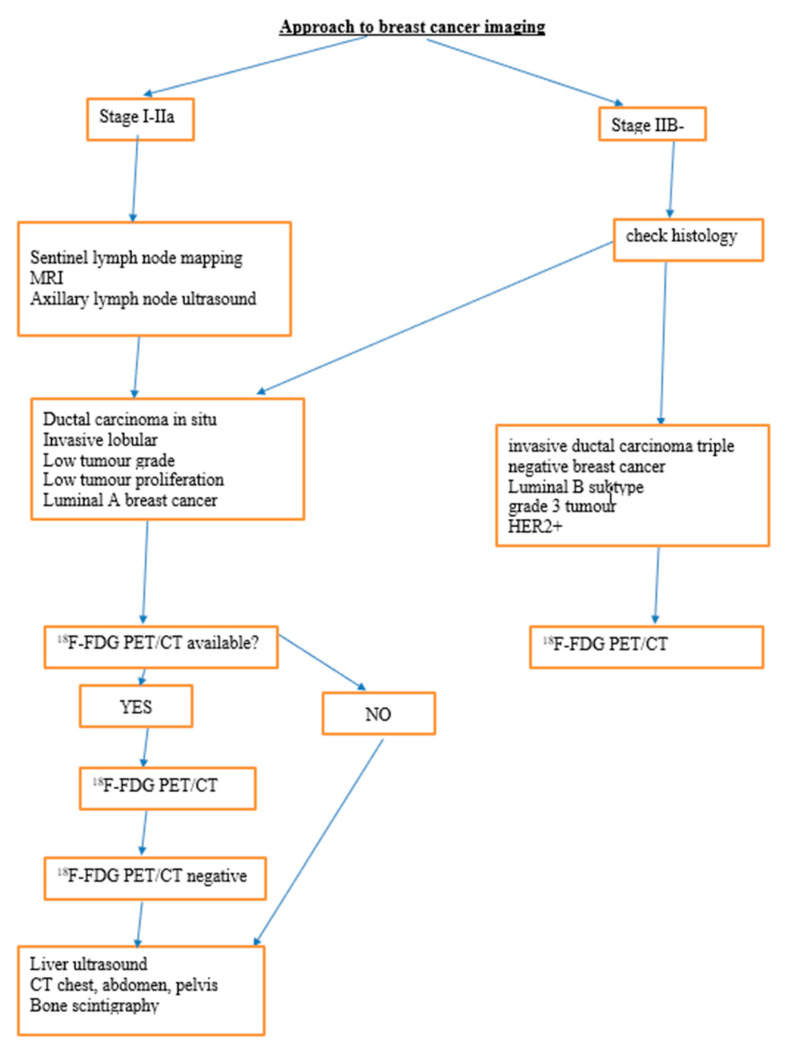
Describes the proposed approach to breast cancer imaging.

In the lung parenchyma, FDG PET is highly sensitive in detecting solid nodules of 10 mm or greater in diameter. PET has a lower sensitivity for smaller nodules due to the partial volume effect and respiratory motion artifacts [12]. The advantage of hybrid PET/CT examination is that small non-FDG avid nodules can be detected on the CT data even though noted the free-breathing CT acquired during hybrid PET/CT is less efficient than standard diagnostic thoracic CT. Moreover, FDG is not specific for malignancy and also accumulates in inflammatory conditions such as TB and sarcoidosis [17]. In the brain, the sensitivity for detecting brain metastasis is low because of high physiologic FDG uptake. Figure 1 shows the recommended approach to breast cancer staging depending on the histopathological subtype of the tumor.

### 2.3. Prognosis

FDG PET/CT has the added benefit of a more accurate prognostic stratification over and above the structural features of the primary tumor assessed by conventional imaging, which plays a crucial role in designing an individualized treatment plan [9]. In general, FDG uptake correlates with tumor aggressiveness and poorer prognosis, and a higher pre-treatment SUVmax predicts poorer outcomes and a higher chance of disease recurrence [9]. In addition, a high SUVmax positively correlates with the tumor size, clinical stage, more aggressive molecular subtypes, and Ki-67 index [27]. Moreover, a negative FDG/PET after chemotherapy predicts better overall survival compared to persistent uptake post-treatment [20]. 

### 2.4. Treatment Response Evaluation

Computed tomography is routinely used to obtain measurements of tumor lesions before and after treatment for response assessment and follow-up [28]. However, functional imaging techniques such as FDG can detect changes in metabolic activity earlier than changes in tumor size as detected by morphologic imaging. This is commonly seen with targeted therapies because such treatments can render tumors metabolically inactive without any substantial modification of their size. 

For endocrine therapy, an increase in tumor FDG uptake 7–10 days after initiating endocrine therapy is predictive of a good response [28]. This phenomenon can be explained by the fact that endocrine therapy has initial agonist effects before antagonist effects dominate. Therefore, an increase in SUVmax in tumors soon after the initiation of hormone therapy is predictive of a good therapeutic response [28,29].

Historically, neoadjuvant chemotherapy (NAC) was used to decrease tumor size and facilitate surgery in locally advanced and irresectable breast cancers. However, recently, its role has evolved to include patients with early-stage resectable breast cancer to down-stage disease to facilitate breast conservation or to avoid axillary nodal dissection by achieving a complete pathological response [30]. Several investigators have found a strong correlation between early changes in FDG maximum SUV and NAC response measured at pathologic examination. They can identify non-responders who need to be switched to other treatment regimens [31]. Zucchini et al. evaluated metabolic changes with FDG PET/CT after receiving NAC in 60 early or locally advanced breast cancer patients, showing that early metabolic non-response was always related to histological non-responders and poor prognosis in ER-positive/HER2-negative patients [29]. This was confirmed in a review article including 745 patients in 15 studies, which showed a moderate pooled sensitivity of 80.5% and specificity of 78.8% of FDG PET for early separation of responders from non-responders could reach after 1 or 2 cycles of NAC [12,21]. Thus, the absence of FDG uptake after therapy predicts better survival rates in patients suffering from metastatic breast cancer. 

Studies of hybrid FDG PET/CT have found that FDG PET was superior to CT and bone scintigraphy in showing response in osseous metastases [14]. FDG PET can detect osseous metastases earlier than CT. Sclerotic lesions appearing at CT and increased uptake on BS after therapy may represent osseous healing rather than new metastases, thus preventing accurate therapy response assessment at CT and BS [32]. A bone scan is also limited in evaluating therapy response in osseous lesions because increasing avidity at the bone scan may represent either increased osseous malignancy or increased osteoblastic response during bone healing after successful therapy [21,23]. This osteoblastic flare response seen at bone scans may persist for several months [33]. Metabolic flare may be seen at FDG PET, which is defined as the apparent worsening of FDG avidity in the first 1–2 weeks after treatment. Thus, it is not a confounding issue on scans that are normally performed months after initiating therapy [8]. It has been found that FDG metabolic flare may be an indicator of future response to therapy [13]. 

### 2.5. Recurrence

Although locoregional recurrence and distant metastasis after the initial treatment carry a poorer prognosis, early detection of the recurrence can improve survival [9]. CT scans, MRIs, and bone scintigraphy are the most commonly used modalities. FDG PET/CT has at least equal accuracy as MRI for detecting locoregional recurrent disease. PET/CT also has high sensitivity and specificity in diagnosing distant metastatic foci. 

In patients with a clinical/biochemical suspicion of recurrence, FDG PET/CT has been found to be useful and compares favorably with other imaging modalities, such as CT, bone scan, or whole-body MRI [14]. A recent study of 100 women with suspected breast cancer recurrence prospectively evaluated the diagnostic accuracy of FDG PET/CT, contrast-enhanced CT, and bone scans. The diagnostic accuracy of FDG PET/CT was better than that of contrast-enhanced CT alone or contrast-enhanced CT combined with bone scan for disease recurrence, with no known false negatives and fewer false positives than the other imaging techniques [23]. 

In a meta-analysis that included 26 studies with 1752 subjects, Xiao et al. showed a pooled sensitivity of 90% and specificity of 81% for PET/CT detection of recurrent breast cancer [34]. Of note, PET/CT is superior to CT and MRI in detecting recurrence because PET screens the whole body in a single session and can also confirm the disease in normal-sized nodes. 

Rising CA 15-3 and CEA levels in asymptomatic patients suggest recurrence, and PET/CT has a high diagnostic yield in detecting recurrence in patients with wising tumor markers. In a retrospective assessment of 228 asymptomatic patients that presented with rising CA 15-3 and/or CEA levels, sensitivity, specificity, PPV, NPV, and accuracy of PET/CT for diagnosing recurrence were 93.6, 85.4, 96.7, 74.5, and 92.1%, respectively [35]. Therefore, PET/CT is recommended in asymptomatic patients with rising CA 15-3 levels or for patients with suspected clinical or radiological recurrence. 

### 2.6. Radiotherapy Planning

For patients undergoing radiation therapy following mastectomy, baseline imaging is vital for radiation therapy planning by defining areas of metabolically active disease that might not be resected at the surgery. In addition, PET/CT detects disease in normal-sized lymph nodes, such as supraclavicular or internal mammary lymph nodes, that may be overlooked on CT alone [36]. Although internal mammary nodes are typically included in the radiation field for patients with inflammatory breast cancer, FDG-PET/CT could allow for tailoring of radiation dose for individual patients and minimize side effects due to irradiation of the heart and lungs. Additionally, the FDG-PET/CT field of view, which typically includes low cervical lymph nodes, has a better yield than the CECT of the chest and abdomen, which typically include cervical nodes [37]. In post-lumpectomy patients, ^18^F-FDG PET–derived volumes tend to be larger than the volumes derived from CT alone.

### 2.7. Positron Emission Mammography

High-resolution breast PET, also known as positron emission mammography (PEM), is a small, organ-specific PET device [38]. In practice, patients are injected with 10–15 millicuries of ^18^F- FDG and imaged 1–3 h after injection [39]. PEM imaging results in a set of 12 slices each in the craniocaudal and mediolateral oblique positions, analogous to mammography [38]. Some users attempt to obtain craniocaudal views of the medial breast [39]. The axilla is often viewable in the mediolateral view [39]. The three-dimensional tomographic image set provides a detailed location of normal and abnormal FDG uptake and features or architectural patterns of any abnormal uptake [38]. 

The technology of PEM and PET are similar in that they both provide functional imaging [38]. However, PEM is optimized for small body parts and utilizes gentle immobilization of the breast to attain higher spatial resolution (1–2 mm for PEM vs. 4–6 mm for PET)*,* as well as minimize the radiation dose by reducing breast thickness [39]. These are seen as some of the benefits of PEM over PET. Other benefits include improved geometric sensitivity with reduced attenuation and shorter imaging time [40].

As both MRI and PEM have similar sensitivities, PEM’s role in clinical practice mirrors that of MRI [38]. Detection and characterization of primary breast lesions in preoperative surgical planning or prechemotherapy evaluation remain primary indications for the exam. Other indications include distinguishing recurrent carcinoma from scar and monitoring response to neoadjuvant chemotherapy [38]. Currently, PEM is used specifically in patients diagnosed with breast cancer considering breast conservation surgery to evaluate for multifocal or multicentric disease [38].

The utility of PEM has been demonstrated in staging both the ipsilateral and contralateral breasts in newly diagnosed patients as an alternative to MRI [41]. MRI has been shown to be more sensitive than PEM in the detection of malignancy, although particularly for ipsilateral lesions, PEM is more specific [41]. Therefore, it can be concluded that in patients in whom MRI may be contraindicated, PEM is valuable in detecting additional foci of malignancy [41].

Despite the high sensitivities described for both exam types, PEM suffers from the same specificity issues as those seen in MRI [38]. The specificity for detecting carcinoma ranges from 85% to 92% for MRI and from 92% to 97% for PEM [38]. A number of non-malignant lesions can accumulate FDG, such as fibroadenoma, fibrocystic change, and fat necrosis [38]. To address this issue, commercially available biopsy systems can be used, allowing vacuum-assisted biopsy of PEM-detected lesions before altering surgical management [40]. Positive predictive values of these biopsies have been similar to those seen for MRI-guided biopsy and higher than that seen for mammography [38]. 

PEM is limited by its high radiation exposure. A single PEM study involving the use of a label-recommended radionuclide dose is associated with a 15-fold higher risk of cancer induction than a single-screen film or digital mammogram [42]. In mammography, fibroglandular tissue is the only tissue exposed to a substantial level of ionizing radiation; however, with PEM, all body organs are irradiated [38]. Therefore, the risk from mammography is essentially only that of induced breast cancer, while PEM can lead to cancer induction in any number of radiosensitive organs [38]. The urinary bladder receives the highest absorbed radiation dose and cancer risk with PEM [38]. 

## 3. Other PET Radiopharmaceuticals in Molecular Imaging of Breast Cancer

PET allows for non-invasive visualization of biological processes in the tumor microenvironment and identification of molecular markers overexpressed in breast cancer, which contributes to early diagnosis and better management of cancer patients. Molecular probes that target metabolism, amino acid transporters, cell proliferation, hypoxia, estrogen receptor (ER), progesterone receptor (PR), human epidermal growth factor receptor 2 (HER2), gastrin-releasing peptide receptor (GRPR), chemokine receptors and fibroblasts have been developed which allow non-invasive detection of the expression of these receptors and the selection of therapeutic targets. The radiotracers for breast imaging other than 18F-FDG are summarized in Table 1.

### 3.1. Fibroblast Activation Protein

**Figure 2:**
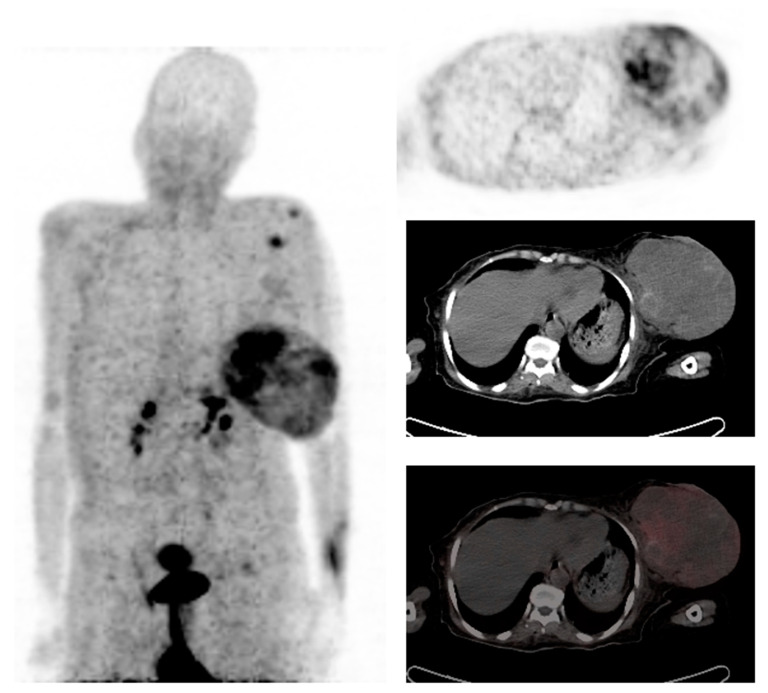
62-year-old female with locally advanced triple negative infiltrating ductal carcinoma of the left breast, post four cycles of chemotherapy. ^68^Ga-FAPI PET demonstrated heterogeneous uptake in the breast primary with tracer-avid shoulder involvement. Images provided by Dr. Janet Reed, Steve Biko Hospital, Pretoria.

Fibroblast activation protein (FAP, FAP-α), a type-II transmembrane serine protease, acts on several hormones and extracellular matrix components and has an essential role in tumor biology [43]. It belongs to the dipeptidyl peptidase 4 family with both post-proline dipeptidyl peptidase and endopeptidase activity [44]. FAP is expressed by cancer-associated fibroblasts (CAFs), and its expression is associated with high tumor proliferation, decreased survival, and worse prognosis in cancer patients [45]. Cancer-associated fibroblasts differ from normal fibroblasts by providing FAP as a target with a relatively high tumor-specific expression [46]. It is also overexpressed in 90% of all epithelial carcinomas, in normal tissue during wound healing, and selectively in benign diseases [47,48]. FAP-targeting imaging is a promising strategy for the visualization of various oncological and non-oncological diseases. It then stands to reason that several FAP-targeting radiopharmaceuticals were developed. Among these is ^68^Ga-FAPI which has a high specificity and affinity for targeting FAP and favorable in vivo pharmacokinetics, as demonstrated in Figure 2.

Studies have revealed that ^68^Ga-FAPI positron emission tomography/computed tomography (PET/CT) imaging clearly delineated tumors and metastases with high tumor-to-background contrast in various tumors [49]. It was noted in a clinical study that in patients with metastasized breast cancer, ^68^Ga-FAPI-04 PET/CT delivered high-contrast images with adequate tracer uptake in metastases and very low uptake in normal tissue [50]. In a study by Komek et al., comparing ^68^Ga-FAPI-04-PET/CT with ^18^F-FDG-PET/CT in 20 female breast cancer patients with primary and recurrent breast cancer, ^68^Ga-FAPI-04-PET/CT had a higher sensitivity than ^18^F-FDG (100% vs. 78.2%) in detecting primary breast lesions [50]. It was also found that ^68^Ga-FAPI-04-PET/CT has an advantage in detecting both primary and metastatic tumors because of its high sensitivity and high SUVmax, as demonstrated in Figure 2 [51]. In a further study by Elboga et al., ^68^Ga-FAPI uptake was observed in primary and metastatic lesions and was statistically significant in pathological breast lesions and lymph nodes [52].

^68^Ga-FAPI-04 is limited by the short half-life of ^68^Ga of 68 min and poor image resolution compared to ^18^F with a half-life of 110 min and its superior image resolution owing to a shorter positron range [53]. As a result, ^18^F-labelled FAPI has been developed, namely ^18^F- FAPI-42 and ^18^F-ALF-FAPI-74, which have shown similar lesion detection to ^68^Ga-FAPI-04 and can be an alternative in areas with poor access to ^68^Ga [54].

### 3.2. Prostate-Specific Membrane Antigen

**Figure 3:**
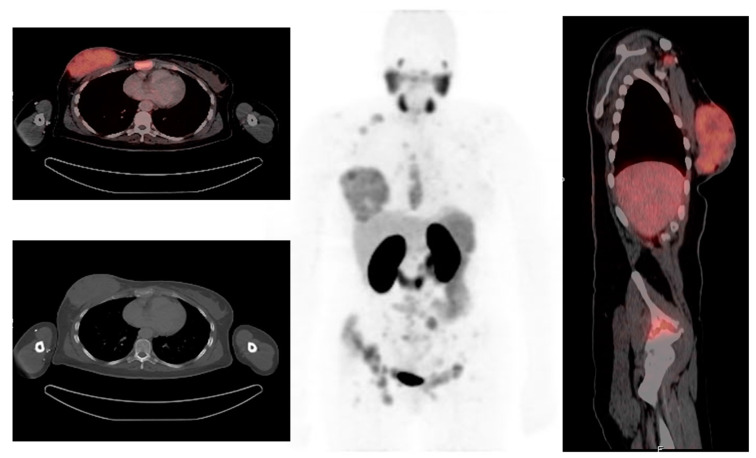
A 39-year-old woman with stage IV breast cancer underwent ^68^Ga-PSMA PET/CT. The Maximum intensity projection PET demonstrated multiple osseous metastases and primary right breast cancer. Axial and sagittal fused PET/CT confirms the ^68^Ga-PSMA avid lesions in the right breast, sternum, and right iliac bone. Images reproduced with permission from [56]; published by Eur. J. Nucl. Med. Mol. Imaging, 2017.

Prostate-specific membrane antigen (PSMA) is an integral membrane protein, mapped to chromosome 11q14, which is over-expressed by a high number of prostate carcinomas and has been reported to be overexpressed in the neovasculature of malignant tumors, including breast cancer, as demonstrated in Figure 3 [55]. In a study by Sathekge et al., ^68^Ga-PSMA-HBED-CC-PET/CT was evaluated in 19 breast cancer patients. A total of 81 lesions were identified, of which 84% were detected by ^68^Ga-PSMA-HBED-CC-PET/CT [56]. In total, seven patients underwent both ^68^Ga-PSMA-HBED-CC and ^18^FDG-PET/CT, with ^18^FDG-PET detecting 35 lesions and ^68^Ga-PSMAHBED- CC-PET detecting 30 lesions. It was noted that six of the ^18^FDG-positive lesions were negative on ^68^Ga-PSMA-HBED-CC-PET, while one of the ^68^Ga-PSMA-HBED-CC-positive lesions was negative on ^18^FDG-PET. In addition, Sathekge et al. suggested that there is a relationship between tumor metabolism as assessed by ^18^FDG uptake and tumor angiogenesis as assessed by ^68^Ga-PSMA-HBED-CC uptake [57]. Therefore, therapies targeting PSMA expression may be an option in patients with breast cancer who are refractory to standard therapies.

### 3.3. Chemokine Receptor 4

**Figure 4:**
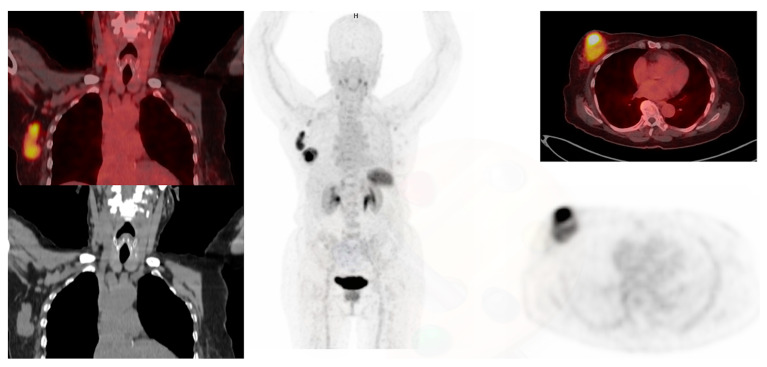
A 74-year-old female with triple-negative breast cancer underwent ^68^Ga-Pentixafor PET/CT. The images demonstrate inhomogeneous CXCR4 receptor expression in the primary lesion in the right breast as well as right axillary lymph nodes.

CXCR4 is a 7-transmembrane G-coupled receptor belonging to the chemokine receptor family and is expressed by various cells during development and thereafter [58]. Its main role in the hematopoietic system is to control stem cell retention and the homing of hematopoietic cells to the bone marrow and lymphoid organs [49]. CXCR4 is frequently overexpressed in invasive breast cancer and has an important role in tumor migration, invasiveness, metastasis, and proliferation [59]. Vag et al. evaluated 18 patients with breast ca who underwent ^68^Ga-Pentixafor PET/CT or PET/MR, 13 of the patients had a first diagnosis of breast cancer, 4 patients had recurrent disease after primary breast cancer, and 1 patient with axillary lymph node metastasis of unknown primary [60]. Sixty nine percent (9/13) of the primary tumors were visually detected with ^68^Ga-Pentixafor, and all 5 metastases could be visually identified. Eight patients (4 recurrent breast cancer patients and 4 primary breast cancer patients) received ^18^F FDG-PET within 2 weeks after administration of ^68^Ga-Pentixafor. It was noted that a higher SUVmax of ^18^F-FDG was observed in all cases, compared with ^68^Ga-Pentixafor. It was also noted that the uptake seen in breast cancer is associated with a poorer prognosis [60]. Higher CXCR4 expression is seen in triple-negative breast cancer compared to the luminal subtypes, as demonstrated in Figure 4. ^68^Ga-Pentixafor PET/CT may have a role in prognostication of breast cancer patients and in selecting potential candidates for therapies targeting CXCR4.

### 3.4. Estrogen Receptor Imaging

Approximately 75% of the tumors express the estrogen receptor (ER) at diagnosis [5,6]. It is important to establish the ER status of a patient as it has important consequences for treatment decision-making because patients with ER-positive tumors are likely to respond to antihormonal therapy [61]. The poor prognostic features in breast cancer include hormone insensitivity, such as lack of estrogen receptor (ER), as well as overexpression of the epidermal growth factor (EGF) family of receptor tyrosine kinases, especially epidermal growth factor receptor (EGF-R, also, HER1 and erbB1) and HER2/neu (erbB2) [5]. Immunohistochemistry staining of the primary tumor is used to establish the hormonal status of the tumor. The primary tumor can have heterogenous expression within it or lose ER expression over time. However, immunohistochemistry is limited by sampling errors and predicts tumor response to antihormonal therapy correctly in only 50–60% of the patients [5]. Moreover, discordant ER expression between primary tumor and metastatic lesions occurs in 18–55% of the patients.

F16a-[^18^F]fluoro-17b-estradiol (^18^F-FES) is an estrogen receptor analog with uptake correlating with estrogen receptor concentration. Its high binding affinity provides clear images of primary and metastatic breast cancer and predicts the effectiveness of endocrine therapy. ^18^F-FES PET leads to better diagnostic understanding in 88% and to a change of therapy in 48% of the patients presenting with a clinical dilemma such as equivocal or conflicting conventional work-up [62]. Patients with positive FES uptake are more likely to benefit and respond to anti-estrogen therapy than those who do not show uptake on FES scans. Therefore, ^18^F-FES PET can be used to assess residual ER availability and eligibility for further hormonal therapy with selective ER downregulators such as fulvestrant. Inadequate reduction of the ^18^F-FES PET signal (<75%) by fulvestrant treatment correlates with early disease progression [63].

### 3.5. Progesterone Receptor Imaging

^18^F-fluorofuranyl norprogesterone (^18^F-FFNP) is a progesterone analog with uptake based on the presence of a progesterone receptor. It provides information on progesterone status in primary and metastatic disease [64]. Progesterone-targeted PET imaging also has the potential to predict response to endocrine therapy [65]. In a study by Dehdashti et al., 43 women with locally recurrent or metastatic ER-positive breast cancer underwent two ^18^F-FFNP scans before and immediately following the one-day estradiol challenge [65]. Twenty-eight patients (65%) responded to treatment and had no disease progression in the 6 months, and all of them showed a post-challenge increase in ^18^F-FFNP uptake in the tumor. In contrast, the remaining 15 patients who progressed within 6 months had no increase in tracer uptake in the tumor [66]. The study demonstrated that the change in ^18^F-FFNP uptake in a tumor after estradiol challenge is highly predictive of responses to endocrine therapy in women with ER-positive breast cancer. Therefore, progesterone-targeted PET imaging with ^18^F-FFNP has the potential to select candidates for endocrine therapy [65].

### 3.6. Human Epidermal Growth Factor Receptor 2 (HER2)

Human epidermal growth factor receptor 2 (HER2) is a member of the family of tyrosine kinase receptors that has an important role in cell growth and survival [67]. Overexpression of the HER2 receptor occurs in approximately 20% to 30% of primary breast cancers and has been associated with relatively poor prognosis, that is, increased recurrence, distant metastasis, and shorter survival [5]. HER2 expression in BC is measured using immunohistochemistry (IHC) detects HER2 overexpression, and fluorescence in situ hybridization (FISH) detects HER2 gene amplification on the pathology specimens. However, there is intra-tumoral and inter-tumoral heterogeneity in HER2 expression, and it is not practical to biopsy every lesion to select the most appropriate therapy and assess the response to therapy.

PET/CT imaging with radiolabeled monoclonal antibodies (mAbs) can be used for non-invasive detection and quantification of specific targets throughout the body and predict the effectiveness of targeted immunotherapies in individual patients [67].

Trastuzumab is an FDA-approved humanized monoclonal antibody that is routinely used as targeted therapy for the treatment of HER2/neu overexpressing breast cancer in combination with other chemotherapy drugs. It targets HER2/neu cancer cells and offers an inhibitory effect on the growth of these cells. Trastuzumab has also been radiolabeled with a number of diagnostic and therapeutic radionuclides such as 64Cu or 68Ga 89Zr to non-invasively assess HER2 expression status in primary breast cancers, lymph node metastases and lung metastases [68]. The advantages of [64Cu]trastuzumab, [68Ga]trastuzumab F(ab′)2 fragments, [68Ga]ABY-002, and [89Zr]trastuzumab PET/CT over biopsy-guided detection of HER2 expression include the ability to assess HER2 expression of the entire tumor volume (which addressing the intrinsic heterogeneity of HER2 expression), directly assessing the binding of the therapeutic mAb (trastuzumab) to HER2, and assessing the response to therapy. Moreover, PET/CT can simultaneously assess HER2 expression of primary and metastatic sites [68]. It has the potential for prognostic information and prediction of response to HER2-targeted therapy.

### 3.7. Androgen Receptor

The androgen receptor (AR) is the most abundantly expressed steroid hormone receptor in breast cancer. It is co-expressed in 75–95% of estrogen receptor (ER)–positive and only 10–35% of triple-negative breast cancers [66]. 16β-[^18^F]fluoro-5α-dihydrotestosterone (^18^F-FDHT) was developed for imaging AR with PET/CT. A study by Venema et al. demonstrated the potential of ^18^F-FDHT and ^18^F-FES PET to serve as non-invasive alternatives to biopsy for detecting metastasis, especially when lesions are difficult to access or sampling errors are prone to occur [69].

In ER-positive breast cancer, AR primarily inhibits tumor proliferation. GTx-024 is a novel oral nonsteroidal elective AR modulation that specifically binds AR-promoting agonist activity. GTx-024 has the advantage of poor binding to other steroidal receptors, no virilizing effects, and it cannot be aromatized with estrogen. In a study by Overmoyer et al., GTx-024 slowed tumor growth in preclinical models of ER-positive breast cancer and was well tolerated [70]. This was confirmed by Jacene et al., who investigated 11 postmenopausal women with estrogen receptor-positive metastatic BC using ^18^F-FDHT PET/CT at baseline and at 6 and 12 weeks after starting SARM therapy with GTx-024 [71]. Even though the small sample size limited the study, they showed clinical benefit in seven participants at 12 weeks. These patients also tended to have larger declines in ^18^F-FDHT uptake than those with progressive disease both at 6 weeks after starting GTx-024 and at 12 weeks after starting GTx-024. These studies show the potential of ^18^F-FDHT PET as an imaging biomarker for evaluating response to selective androgen receptor modulator (SARM) therapy.

### 3.8. Somatostatin Receptor Expression

**Figure 5:**
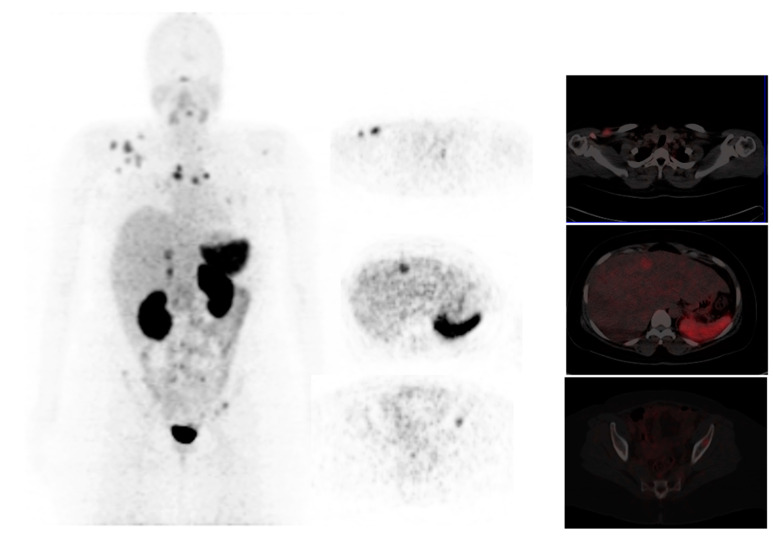
A 39-year-old female with right breast invasive ductal carcinoma, PR negative and ER positive. She underwent a mastectomy, radiation therapy, chemotherapy, and hormonal therapy and was referred for a restaging PET/CT. ^68^Ga-DOTATATE imaging demonstrated metastatic involvement of the mediastinal- and axillary lymph nodes, lungs, liver- and skeletal system. Images provided by Dr. Janet Reed, Steve Biko Hospital, Pretoria.

Somatostatin receptors (SSTR) are variably expressed in primary breast cancer tumors, and there is a positive correlation between several receptor subtypes (SSTR1, SSTR2, and SSTR4) and hormone receptor (HR) positive tumors [72]. Breast tumors expressing hormone receptors (ER, PR) have significantly lower FDG uptake than tumors that do not express HR. Breast cancers are known to demonstrate avidity on SSTR imaging, as shown in Figure 5. Nguyen et al. investigated 10 patients with ER+, PR+, and HER2- breast cancer patients with ^68^Ga-DOTATATE and ^18^F-FDG PET/CT and compared the findings with conventional imaging (bone scan and diagnostic CT) [73]. The total lesion detection rate of DOTATATE was comparable to FDG and conventional imaging for primary breast tumors and nodal and bone metastases; however, DOTATATE demonstrated a lower detection rate of visceral lesions compared with FDG. ^68^Ga-DOTATATE demonstrated higher uptake in 1 ER+ patient that underwent biopsy compared to FDG. Therefore STSR imaging may have a role in patients with poor FDG uptake and guide hormonal therapy. However, a study comparing ^68^Ga-DOTATATE with ^18^F-FES for this indication is warranted.

### 3.9. Integrins

The presence of angiogenesis is one of the predictors of poor prognosis in breast cancer, an increased level of angiogenic growth factors in the breast cancer cells correlates with the aggressiveness and risk of invasive breast cancer [74]. Furthermore, the number of microvessels in an invasive breast carcinoma from surgical samples may be a predictor of metastasis or relapse. Integrin avb3 is one of the most important members of the integrin family and plays a vital role in the regulation of cellular activation, survival, and migration. PET/CT imaging provides the ability to visualize and quantify avb3 integrin expression using specific targeting ligands to evaluate tumor neovascularization and identify patients with potentially more aggressive diseases [75].

Cyclic RGD peptides have high affinity and selectivity for integrin avb3. Therefore various arginine-glycine-aspartic acid (RGD)–containing peptide probes have been tested, such as ^18^F-AlF-NOTA-PRGD2, denoted as ^18^F-alfatide. Wu et al. compared ^18^F-alfatide with ^18^F-FDG in 42 patients with histologically proven breast cancer and 11 benign breast lesions [75] and found the two tracers to be complementary [76]. Individually both radiotracers had high sensitivity (88.1% vs. 90.5%), high positive predictive value (88.1% vs. 88.4%), moderate specificity (54.5% vs. 54.5%), and moderate negative predictive value (54.5% vs. 60.0%) for differentiating breast cancer from benign breast lesions. By combining ^18^F-alfatide and ^18^F-FDG, the sensitivity and negative predictive value significantly increased.

The anti-VEGF antibody (bevacizumab), which inhibits the VEGF pathway, has been established as an antiangiogenic treatment in non-small cell lung, colorectal, and breast cancer [74]. Kazmierczak used ^68^Ga-TRAP (RGD)3 PET/CT for monitoring in vivo αvβ3-integrin expression in breast cancer xenografts in mice treated with bevacizumab over the course of 1 week [76]. RGD uptake in animals treated with bevacizumab was decreased subsequent to VEGF inhibition, whereas it remained the same in the untreated group. ^68^Ga-TRAP (RGD)3 has a role in the selection of patients who may benefit from the therapies targeting angiogenesis, monitoring treatment response, and can potentially predict prognosis [76].

### 3.10. Gastrin Releasing Peptide Receptor

GRPR is a subtype of the bombesin receptor family with the physiologic ligand gastrin-releasing peptide (GRP) [77]. GRP has various physiologic functions, including the release of gastrin and regulation of enteric motor function. GRP and gastrin-releasing peptide receptor (GRPR) also appear to play a role in human carcinogenesis and tumor proliferation. Breast cancers express gastrin-releasing peptide (GRP) hormone and gastrin-releasing peptide receptor (GRP-R), and its expression is associated with lymph node metastases. Stovkow et al. evaluated 15 female patients with biopsy-confirmed primary breast carcinoma with ^68^Ga-RM2-PET/CT for pre-treatment staging [78]. In vivo tumor uptake of ^68^Ga-RM2 was correlated with estrogen (ER), progesterone (PR) receptor expression, HER2/neu status, and MIB-1 proliferation index in breast core biopsy specimens. Higher uptake was seen in ER-positive compared to ER-negative tumors. Moreover, ^68^Ga-RM2-PET/CT was superior to ^18^F-FDG, which is limited by non-specific uptake at sites of inflammation/infection and inability to detect small lymph node metastasis, ^68^Ga-RM2-PET/CT has a higher target-to-background ratio due to very low radiotracer uptake in muscles and fat tissue, which allows for better visualization of affected lymph nodes less than 5 mm in maximum diameter.

^68^Ga-RM2-PET/CT may also have an impact on patient management, showed a high detection rate for suspicious internal mammary lymph nodes (IMLN) (53%; 8/15), leading to potential upstaging of 40% of the patients (6/15). GRPR targeting also provides a potential for a new therapeutic approach via peptide receptor radionuclide therapy (PRRT) in patients with GRPR-positive BC [78]. GRPR antagonists, such as RM2, labeled with a therapeutic radioisotope (e.g., Lutetium-177 or Yittrium-90) could be used to treat BC patients while using Ga-68 labeled RM2 as a diagnostic companion to select potential candidates for this therapy and monitoring treatment response.

### 3.11. PARP Inhibitors

Poly(ADP-ribose) polymerase-1 (PARP-1) is a key enzyme in the DNA repair process, and the overexpression of PARP-1 in several tumours makes this enzyme a promising molecular target [79]. PARP inhibitors inhibit the catalytic activity of PARP-1 and trap PARP-1 on damaged DNA resulting in conformational changes of PARP-1, which promotes cell apoptosis. In 2014, several PARP-1 inhibitors, namely olaparib, rucaparib, niraparib and talazoparib, were clinically approved as anticancer drugs by the FDA. Subsequent to that, two of the radiolabelled olaparib and rucaparib analogues (^18^F-PARPi and ^18^F-FTT) have entered clinical trials with applications to breast cancer as well as brain tumours, and ovarian cancer. 

Accumulating evidence suggests that further clinical exploration of PARPi as monotherapy or combinations have shown benefit in patients with BRCA1 or BRCA2 mutation (gBRCAm)-associated breast cancer, as well as in breast cancer with homologous recombination repair (HRR) dysfunction [80]. Imaging of PARP therefore has a potential role in selecting patients for treatment with PARPi and monitoring treatment response. 

### 3.12. Hypoxia Imaging

Hypoxia occurs in breast cancer and in other solid tumours due to the tumour outgrowing the existing vasculature. Tumor hypoxia increases metastatic potential in breast cancer and is a strong prognostic factor of disease progression and survival [81]. Additionally, rapid tumor growth can cause increased consumption of oxygen, along with poor formation of vasculature which impedes sufficient oxygen delivery. Hypoxic tumor cells show resistance to radiation therapy, targeted therapies, and chemotherapy [81] and decreases the growth promoting effects of estradiol and growth inhibitory effects of anti-oestrogen therapy in ER+ breast cancer lines. 

**Figure 6:**
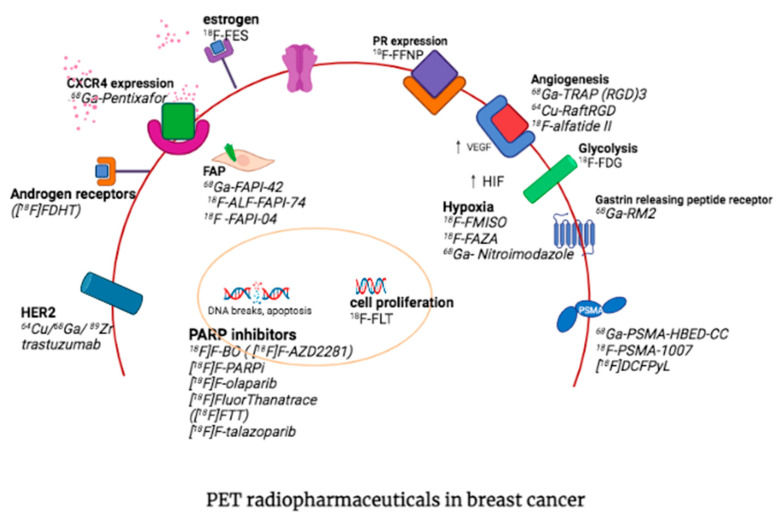
Shows the various PET tracers that target breast cancer and their site of action on the tumor cells.

^18^F-fluoromisonidazole (FMISO) positron emission tomography (PET) with computed tomography (CT) is the most widely accepted imaging technology available for the localization and quantification of intracellular hypoxia in vivo [81]. FMISO selectively accumulates in viable hypoxic cells but not in necrotic cells and normoxic cells. Accumulation of FMISO on PET/CT, has been shown to correlate with a shorter DFS in patients with primary breast cancer. In a study by Asano et al., triple-negative breast cancer demonstrated a significantly higher FMISO-TBR than luminal A and the FMISO-TBR was significantly correlated with larger tumour size, higher nuclear grade, and negative oestrogen receptors and progesterone receptor. The authors concluded that FMISO-PET/CT noninvasively provides hypoxic information and helps identify patients with a baseline risk of early recurrence and those eligible for antiangiogenic therapy, regardless of size, nuclear grade, and nodal metastasis [81]. Figure 6 summarises the the various PET tracers that target breast cancer and their site of action on the tumor cells.

### 3.13. PET/CT Imaging of Bone Metastasis

#### 3.13.1. ^18^F-NaF Bone Imaging

The increased availability of positron emission tomography (PET) and hybrid PET/computed tomographic (PET/CT) systems and the sporadic availability of ^99m^Tc have led to the revival of ^18^F-NaF for osseous imaging [82]. ^18^F-NaF has a similar action mechanism to ^99m^Tc-MDP, based on ion exchange with hydroxyl ions on the outside of the hydroxyapatite that converts hydroxyapatite to fluorapatite. ^18^F-NaF has the better image quality and shorter ^18^F-NaF imaging time owing to its pharmacokinetic properties, such as higher osseous uptake and faster blood clearance of ^18^F-NaF due to less protein binding. Similar to ^99m^TcMDP, ^18^F-NaF is limited by low specificity for ruling out metastatic skeletal involvement.

When compared to ^18^F-FDG, ^18^F-NaF is more sensitive for the detection of bone metastasis in breast cancer; however, ^18^F-FDG PET detects extra-skeletal disease that can significantly change disease management [83]. However, ^18^F-FDG PET/CT has limitations in detecting osteoblastic skeletal lesions. ^18^F-FDG PET/CT has a higher sensitivity than BS, especially for the detection of lytic lesions (sensitivity up to 100%) and of metastatic cells still confined within the bone marrow, before the occurrence of the cortical osteoblastic reaction required for identification at BS.

In addition, ^18^F-FDG PET/CT is independently associated with overall survival in breast cancer patients with bone metastases. The complementary role of bone scintigraphy and ^18^F-FDG PET/CT has led to the investigation of ^18^F-FDG PET/CT and ^18^F-NaF PET/CT cocktail [84]. In a study by Roop et al. comparing ^18^F-FDG PET/CT with a cocktail of ^18^F-FDG PET/CT and ^18^F-NaF PET/CT in 70 patients with locally advanced breast cancer *n* = 50 (71.0%), and recurrent breast cancer (*n* = 20), the cocktail was superior to ^18^F-FDG PET/CT alone for the detection of skeletal/marrow metastases in breast cancer [84]. In eight patients (11.4%), only cocktail PET/CT identified skeletal/marrow lesions, whereas ^18^F-FDG PET/CT was negative. Therefore, cocktail PET/CT impacted the management of these eight patients because of upstaging of disease. The only drawback is that cocktail PET imaging makes it impossible to determine whether a site of pathological uptake is attributed to ^18^F-NaF or ^18^F-FDG, thereby losing prognostic information carried out by ^18^F-FDG in skeletal sites [85].

#### 3.13.2. ^68^Ga- Zoledronate

Gallium-68 zoledronate (^68^Ga-DOTA^ZOL^) has been proposed to be a potent bisphosphonate for PET/CT diagnosis of bone diseases and has shown high and selective uptake in bone lesions [83]. The possibility of treatment of bone metastases with ^177^Lu-DOTA^ZOL^ and ^225^Ac-DOTA^ZOL^ gives it a clear advantage over other bone-seeking radiotracers such as ^18^F Na-F and ^99m^Tc-MDP [86]. In a study by the Pretoria group, ^68^Ga-PSMA-11 PET/CT detected more lesions than ^68^Ga-NODAGA^ZOL^ PET/CT and ^99m^Tc-MDP bone scan for the staging of skeletal metastases. However, the authors suggest that ^68^Ga-NODAGA^ZOL^ has a role in patients with PSA progression on PSMA-based radioligand therapy, where ^68^Ga-NODAGA^ZOL^ PET/CT is a more appropriate imaging modality for the detection of skeletal lesions not expressing PSMA [87].

## 4. Targeted Therapies in Breast Cancer

### 4.1. FAPI

In a case report by Ballal et of a 31-year-old female with metastatic ER-, PR- and HER 2+ breast cancer with disease progression on standard lines of therapy and intense radiotracer accumulation was noted in all the lesions on ^177^Lu-DOTATATE dosimetry images in concordance to ^68^Ga-DOTA-FAPi PET/CT scans [88]. Lindner et treated one patient with metastatic breast cancer with 2.9 GBq of ^90^Y-FAPI-04 [89]. The bremsstrahlung images showed accumulation of the tracer at 3 h and even at 1 d after injection in this patient. Despite the low dose administered, there was a significant reduction in pain medication use post-therapy. Baum administered ^177^Lu-FAPI in 11 patients with solid tumors, four of which had breast cancer. Biodistribution images after therapy revealed significant uptake of ^177^Lu-FAP-2286 and long retention of the radiopharmaceutical in all patients, FAPI-02 and FAPI-04, revealed an earlier ^177^Lu-FAPI washout and a correspondingly shorter retention time [90]. In one breast cancer patient, ^68^Ga-FAP-2286 PET/CT revealed a mixed response post-therapy (i.e., remission of the diffuse bone metastases). Still, the overall disease was progressive, with evidence of new hepatic lesions. Another breast cancer patient demonstrated progression at 8 weeks after the third cycle of FAPI-targeted radionuclide therapy. This initial experience highlights the safety of ^90^Y-FAPI-04 and ^177^Lu-DOTA.SA.FAPI and the potential for its use in patients who are refractory to standard therapies.

### 4.2. CXCR4 Antagonists

Although new treatments are emerging, no established standard of care exists for HER2-negative patients with relapsed metastatic breast cancer, particularly for patients with hormone receptor-positive metastatic breast cancer after their disease becomes refractory to hormone therapies [91]. A few preclinical studies have demonstrated the ability of CXCR4-targeted therapies to inhibit cancer progression and metastasis in breast cancer [92,93]. In addition, it has been demonstrated that therapies targeting CXCR4 may have a role in Trastruzumab refractory disease [94].

Balixafortide is a potent, selective antagonist of CXCR4 with a high affinity for human CXCR4 receptor shown to be used to chemosensitize tumor cells to eribulin through the disruption of the SDF-1- mediated prosurvival signaling in the tumor microenvironment in preclinical studies. Balixafortide enhances the cytotoxic effect of chemotherapeutic agents and is being investigated in metastatic breast cancer [91].

In a phase 1 single-arm trial in 56 heavily pre-treated patients with relapsed HER2-negative metastatic breast cancer, Pernas et al. showed that the combination of balixafortide and eribulin was safe and was tolerated well [91]. This was confirmed in a phase 3 randomized multicenter FORTRESS trial [92]. Objective responses were seen in 16 (35%) of 54 patients, who were all partial responses. However, the efficacy was not different in the two arms in the FORTRESS trial.

There is no consensus currently regarding the efficacy of CXCR4 antagonists in TNBC. Zhou et al. demonstrate that the CXCR4 antagonist AMD3100 has been shown to increase sensitivity to radiation therapy in triple-negative breast cancer tumor models [95]. In contrast, Lefort et al. argue that knocking the CXCR4/CXCL12 pathway with AMD3100 and TN14003 does not reduce tumor growth and can even increase tumor spread in TNBC [96] AMD3100 reverses tamoxifen resistance by decreasing phosphorylated (p)-AKT levels of tamoxifen-resistant cells and a combination of Tamoxifen and AMD3100 could be efficacious in the treatment of tamoxifen resistance [97].

### 4.3. ^177^Lu-Trastuzumab

Bhusari et al. evaluated seven patients with metastatic breast cancer (HER 2 positive *n* = 5) and HER2 negative disease (*n* = 2) with a low dose of ^177^Lu-trastuzumab (10 mCi) [98]. The images showed localization in primary and metastatic lesions, specifically in histopathology-proven HER2-positive patients. No tracer uptake could be observed on planar and SPECT/CT imaging of the HER2-negative patient. The authors recommend that imaging is performed on day 5/7, post administration of ^177^Lu-trastuzumab, to allow for clearance of background activity. ^177^Lu-trastuzumab proved effective in targeting HER2-positive breast cancer lesions with great specificity and was a potential palliative agent for radioimmunotherapy in HER2-positive metastatic breast disease [98,99]. This study shows the potential for palliative ^177^Lu-trastuzumab in patients who are refractory to standard therapy and those who develop resistance to conventional therapies.

## 5. Concluding Remarks and Future Perspectives

PET/CT imaging with ^18^F-FDG has superior diagnostic efficacy compared to conventional morphological imaging for detecting regional and distant metastasis in breast cancer. Additional findings from FDG PET/CT have a significant impact on therapeutic plans and result in a change in initial staging and affects therapeutic management, that is, upstage patients and leads to the omission of neoadjuvant chemotherapy and surgery in patients with stage IV disease, not detected on conventional imaging. Therefore, ^18^F-FDG PET/CT should replace conventional imaging in patients with clinical stage IIb-III breast cancer. This will lead to more accurate staging and avoidance of adverse effects and the cost of unwarranted neoadjuvant chemotherapy surgery and radiation therapy in patients with disseminated disease. In addition, ^18^F-FDG PET/CT can identify patients with oligometastasis who may benefit from local ablative therapies such as metastasectomy, and stereotactic body radiotherapy, which prolongs survival in these patients. Moreover, FDG PET/CT is superior to conventional imaging for identifying extra-axillary nodal metastasis and influences planning fields for surgery and radiation therapy. In centers with limited PET/CT availability, ^18^F-FDG PET/CT should be performed in patients whose imaging is suspicious but not diagnostic of metastasis.

^18^F-FDG is not without limitations and has low specificity due to its accumulation in non-malignant disease processes such as sites of infection, inflammation, and gastrin-releasing peptide receptor imaging (^68^Ga-RM2) has been shown to be superior to FDG in this regard. More research needs to be done in this area. In addition, FAP imaging with ^68^Ga FAPI-42 imaging shows a superior target-to-background ratio, especially in the brain where FDG is limited by high physiological uptake. These novel tracers also have the potential for targeted therapies in patients who are refractory to standard therapies. Future research should also look into the impact of these new targets in patients where FDG is limited, such as low-grade/low proliferation tumors, invasive lobular or ductal carcinoma in situ, and luminal A histology, as these have been shown to have low uptake on FDG PET imaging.

**Table 1:** PET tracers for breast cancer beyond ^18^F-FDG.

PET Tracers	Class	Biochemical Mechanism	Clinical Application	Level of Evidence
^68^Ga-PSMA-HBED-CC ^18^F-PSMA-1007 [^18^F]DCFPyL	Prostate-specific membrane antigen	PSMA inhibitors angiogenesis	Staging Potential for treatment response monitoring [56,57,100]	Systematic review
^18^F-FLT (flurothymidine)	Cell proliferation	Substrates for cytosolic thymidinekinase-1 (TK1), which catalyzes the initial metabolic step of thymidine triphosphate synthesis.	Staging, monitoring, and prediction of response to treatment Uptake correlates with proliferation index ki-67 [101,102]	Systematic review and meta-analysis
^−11^C-choline or ^18^F-choline	Membrane Lipid Synthesis	Intracellular phosphorylation by choline kinase to phosphorylcholine. Associated with phospholipids of the cell membrane and tumor growth.	Assessment of tumor progression and Monitoring response to therapy [103,104]	Peer review
^11^C-methionine ^18^F-Fluciclovine	Amino Acid Transport	Uptake related to amino acid transport in tumor cells	Assessment of disease, response to therapy and distinguishing responders from non-responders [104]	Peer review
^68^Ga FAPI-42 ^18^F-ALF-FAPI-74 ^18^F-FAPI-04	Fibroblast activation protein	Overexpressing fibroblast activation protein by cancer-associated fibroblasts (CAFs)	Staging Monitoring response to therapy Potential for selection of treatment response [88,89,90]	Peer review
F16a-[18F]fluoro-17b-estradiol (^18^F-FES)	Estrogen receptor imaging	Establish the ER status	Non-invasive detection of ER status in primary and metastasis Select candidates for anti-estrogen therapy [61,62,63]	Peer review
[^18^F]-fluorofuranyl norprogesterone ([^18^F]FFNP)	Progesterone receptor imaging	Progesterone analogue	Non-invasive detection of PR status predict response to endocrine therapy [64,65]	Peer review
16β-[^18^F]fluoro-5α-dihydrotestosterone ([^18^F]FDHT)	Androgen receptor	Testosterone analog	Non-invasive alternative to biopsy imaging biomarker for evaluating response to SARM therapy [69,70,71]	Peer review
^64^Cu trastuzumab ^68^Ga trastuzumab ^89^Zr trastuzumab	HER-2 receptor	Humanized monoclonal antibody	Prognostic information assessing the expression of HER2 in tumors prediction of response to HER2 targeted therapy [67,68,99]	Peer review
^68^Ga-DOTATATE	Somatostatin receptor expression	Somatostatin receptor analog	May have a role in ER+ and PR+ breast cancer with low FDG uptake Lower detection of visceral lesions than FDG [72,73]	Peer review
^68^Ga-TRAP (RGD)3 ^64^Cu-RaftRGD ^18^F-alfatide II	Integrin alpha v beta (RDG)	Angiogenesis	Complementary to FDG PET Select patients who may benefit from therapies targeting angiogenesis Monitor treatment response Prognosis [74,75,76]	Peer review
^68^Ga-RM2	Gastrin-releasing peptide receptor	Overexpression of the physiologic ligand gastrin-releasing peptide in breast cancer	Assess disease extentPotential for selecting candidates for GRPR antagonists Superior to FDG (less uptake in inflammation, infection, and background) [77,78]	Peer review
[^18^F]F-BO (also known as [^18^F]F-AZD2281 [^18^F]F-PARPi [^18^F]F-olaparib [^18^F]FluorThanatrace ([^18^F]FTT) [^18^F]F-talazoparib	PARP inhibitors	Blocking the repair pathway of DNA double-strand breaks and promoting cell apoptosis	Patient selection for treatment with PARPi treatment monitoring [79,80]	Peer review
^18^F-FMISO ^18^F-FAZA ^18^F-FETNIM ^18^F-HX4 ^60/64^Ga-ATSM ^68^Ga- Nitroimidazole	Hypoxia	Selective accumulation in viable hypoxic cells	Non-invasively provides hypoxic information [81] Helps identify patients with a risk of early recurrence Identify patients eligible for antiangiogenic therapy [87,88]	Peer review
^8^F-sodium fluoride ^(18^F-NaF)	Fluoride	ion exchange with hydroxyl ions on the outside of the hydroxyapatite that converts hydroxyapatite to fluorapatite	Detection of bone metastasis [79,82]	Peer review
^68^Ga- Zoledronate	Bisphosphonate	Accumulates in areas of high bone turnover	Detection of bone metastasis Selection for [^177^Lu]Lu-DOTA^ZOL^ and [^225^Ac]Ac-DOTA^ZOL^ [86,87]	Peer review

## Abbreviations

BC	breast cancer
CEA	carcinoembryonic antigen
DCIS	ductal carcinoma in situ
HER 2+	Human Epidermal growth factor Receptor 2 oncogene
IBC	Inflammatory breast cancer
SARM	nonsteroidal elective AR modulation
LS	lymphoscintigraphy
MDP	methylene diphosphonate
NCCN	national embryonic cancer network
SLN	sentinel lymph node
SLNB	sentinel lymph node biopsy
TNBC	triple-negative breast cancer
